# Proton Radiotherapy for Vestibular Schwannomas in Patients with NF2-Related Schwannomatosis: A Case Series

**DOI:** 10.3390/curroncol30030263

**Published:** 2023-03-20

**Authors:** Jules P. J. Douwes, Kimberley S. Koetsier, Victor S. van Dam, Scott R. Plotkin, Frederick G. Barker, D. Bradley Welling, Jeroen C. Jansen, Erik F. Hensen, Helen A. Shih

**Affiliations:** 1 Department of Otorhinolaryngology—Head and Neck Surgery, Leiden University Medical Center, 2333 ZA Leiden, The Netherlands; 2 Department of Otorhinolaryngology—Head and Neck Surgery, Erasmus MC, 3015 GD Rotterdam, The Netherlands; 3 Department of Neurology, Massachusetts General Hospital, Harvard Medical School, Boston, MA 02114, USA; 4 Department of Neurosurgery, Massachusetts General Hospital, Harvard Medical School, Boston, MA 02114, USA; 5 Department of Otorhinolaryngology—Head and Neck Surgery, Massachusetts Eye and Ear Infirmary, Harvard Medical School, Boston, MA 02114, USA; 6 Department of Radiation Oncology, Massachusetts General Hospital, Harvard Medical School, Boston, MA 02114, USA

**Keywords:** acoustic neuroma, cranial nerve, hearing, neurofibromatosis type 2, proton radiotherapy, schwannomatosis, toxicity, vestibular schwannoma

## Abstract

**Simple Summary:**

The management of vestibular schwannomas in neurofibromatosis type 2-related schwannomatosis (NF2) is complex. Current treatment strategies include active surveillance, surgery, radiotherapy, and pharmacotherapy. Due to treatment side effects such as hearing loss, balance complaints, or cranial nerve deficits, it remains challenging to find a treatment that achieves a good control of tumor growth with minimal side effects. The present study found that the majority of NF2 patients was treated with proton radiotherapy as salvage treatment for vestibular schwannoma and did not require additional interventions. However, they did experience significant tumor and/or treatment-related side effects. The value of proton radiotherapy as a primary treatment for vestibular schwannomas in NF2 patients remains to be determined.

**Abstract:**

(1) Background: This study aimed to evaluate the efficacy and treatment-related toxicity of proton radiotherapy (PRT) for vestibular schwannoma (VS) in patients with neurofibromatosis type 2-related schwannomatosis (NF2). (2) Methods: Consecutive NF2 patients treated with PRT for VS between 2004 and 2016 were retrospectively evaluated, focusing on tumor volume, facial and trigeminal nerve function, hearing, tinnitus, vestibular symptoms, and the need for salvage therapy after PRT. (3) Results: Eight patients were included (median age 36 years, 50% female). Median follow-up was 71 months. Five (63%) patients received fractionated PRT and three (38%) received PRT radiosurgery for VS. Six patients (75%) received prior VS surgery; three also received bevacizumab. Six patients (75%) did not require salvage therapy after PRT. Two patients (25%) with residual hearing lost it after PRT, and six had already lost ipsilateral hearing prior to PRT. Tumor and treatment-related morbidity could be evaluated in six patients. Following PRT, conditions that occurred or worsened were: facial paresis in five (83%), trigeminal hypoesthesia in two (33%), tinnitus in two (33%), and vestibular symptoms in four patients (67%). (4) Conclusion: After PRT for VS, the majority of the NF2 patients in the cohort did not require additional therapy. Tumor and/or treatment-related cranial nerve deficits were common. This is at least partly explained by the use of PRT as a salvage treatment after surgery or bevacizumab, in the majority of cases. There remains the further opportunity to elucidate the efficacy and toxicity of proton radiotherapy as a primary treatment.

## 1. Introduction

Neurofibromatosis type 2-related schwannomatosis (NF2) is an autosomal dominant tumor syndrome caused by mutations in the NF2 gene on chromosome 22q12.2. It predisposes to the development of multiple benign tumors such as schwannomas, meningiomas, and ependymomas. The hallmark lesion of NF2 is a bilaterally occurring vestibular schwannoma (VS). Typically, VS presents with hearing loss, balance disturbances, and tinnitus. Rarely, VS may cause facial nerve paralysis and trigeminal nerve hypoesthesia, especially with larger tumors. If left untreated, VS may eventually lead to potentially fatal elevation of intracranial pressure or brainstem compression. Compared to sporadic VS, NF2-related VS arises at a younger age, almost always occurring bilaterally, and is often polyclonal, meaning that the tumor is comprised of several, distinct benign masses. Patients with NF2 typically present with multiple and larger tumors, which exhibit a higher growth rate and respond poorly to standard therapies, such as surgical resection and photon radiotherapy (PhRT). Consequently, NF2-related VS leads to a higher morbidity and reduced quality of life (QoL) compared to sporadic VS [1,2,3,4,5,6]. The clinical presentation and severity of NF2 is highly heterogenous. Patients can be divided into two main phenotypic groups, a severe (Wishart) type characterized by the development of multiple progressive cranial and spinal schwannomas, meningiomas, and ependymomas from childhood, and a milder (Feiling–Gardner) type, characterized by a later age of onset and, usually, a limited number of slowly progressive tumors, often only bilateral VS [7,8].

The management of VS in NF2 is challenging. Current strategies include active surveillance, surgery, PhRT, and pharmacotherapy with bevacizumab [9,10,11]. Photon radiotherapy achieves a high tumor control, either as fractionated PhRT or single fraction stereotactic radiosurgery (SRS), but side effects, such as decreased hearing, vestibular complaints, or the increased impairment of facial nerve and trigeminal nerve function, are not uncommon [12,13]. Therefore, it remains challenging to find a treatment that achieves good tumor control with minimal treatment-induced side effects. Proton radiotherapy (PRT) may be an interesting alternative to PhRT due to the distinct physical properties of protons. A highly localized energy deposition in a target region, known as the Bragg peak, allows high-dose irradiation of tumor tissue with minimal to moderate low-dose irradiation risk to the surrounding brain, brainstem, cochlea, and cranial nerves like the facial and trigeminal nerves [14,15]. A theoretical treatment-planning study recently compared PhRT to PRT for skull-base meningiomas. They found that PRT achieved a considerable radiation dose reduction administrated to healthy brain tissue, such as the hippocampi [16]. Especially in a tumor-predisposing syndrome like NF2, a reduction of the radiation dose could be important as patients are genetically prone to malignant transformation of irradiated tumors or the occurrence of new primary tumors. As reports on VS treatment with PRT in NF2 patients are scarce, it is currently unclear whether PRT can reduce treatment-related toxicities for NF2 patients [17,18,19,20].

The present case series aims to evaluate the treatment-related outcomes and toxicity in NF2 patients who were treated for VS with PRT at a single institution.

## 2. Materials and Methods

After approval by the institutional review board from Mass General Brigham, charts of NF2 patients consecutively treated with PRT for VS between 2004 and 2016 at the Francis H. Burr Proton Therapy Center in the Department of Radiation Oncology, Massachusetts General Hospital (Boston, MA, U.S.A) were retrospectively reviewed. The inclusion criteria were a clinically and radiologically confirmed diagnosis of NF2, according to the 1997 Manchester criteria, and the presence of at least one VS treated with PRT [21]. The diagnosis of vestibular schwannoma was made by the referring clinician according to local protocols, using either the MR image and/or the histopathology in the case of surgical resection prior to PRT. The exclusion criteria consisted of patients with a unilateral or sporadic VS, VS as part of another syndrome, and the absence of follow-up data.

At our center, radiotherapy is not the first option for NF2 patients. If intervention is indicated, initial treatment with bevacizumab will generally be attempted to control tumor progression and preserve hearing. If bevacizumab fails, or is not well tolerated, surgery is considered generally before radiation therapy. Surgical intervention may also be preferred in the case of a large VS or imminent complications like brainstem compression. Radiotherapy is often considered in older patients, or when bevacizumab and/or surgery have failed or are not feasible. As a consequence, NF2 patients who are indicated for active treatment of their VS have often received either bevacizumab, surgery, or both, prior to treatment with radiotherapy. The decision to use PRT (over PhRT) as the radiation modality was based on the accessibility of radiation techniques at the time of treatment, patient preference, and the insurance reimbursement policy. Proton radiotherapy was delivered with a cyclotron-based passive scattering system. The prescribed dose was 50.4 to 54 Gy (relative biological effectiveness: RBE) in 28 or 30 fractions, or 12 Gy (RBE) in proton SRS. While there is no golden standard on dosimetry for PRT in VS, our center made use of universally accepted treatment plans [22].

Baseline and follow-up evaluations consisted of clinical, radiological, and audiometric assessment. Pre- and post-treatment outcomes of interest included tumor volumetrics, the need for salvage therapy targeted at the VS which was treated with PRT during follow-up, facial nerve and trigeminal nerve function, hearing, tinnitus, and vestibular symptoms. Tumor dimensions were assessed before and after PRT in cubic centimeters (cm^3^) derived from the Magnetic Resonance Imaging (MRI) using the REiNS criteria [23]. The audiometric testing included pure tone audiometry (PTA) and a word recognition score (WRS) acquired with the W22 word list. As per the recommendation by the American Academy of Otolaryngology–Head and Neck Surgery (AAO-HNS), the PTA represents the average hearing loss at 0.5, 1, 2, and 3 kilo Hertz (kHz) [24]. Serviceable hearing was defined as a PTA threshold < 50 decibels (dB) and a WRS ≥ 50% [25], otherwise classified as class B hearing [24]. Residual hearing was defined as a PTA < 100 dB and a WRS < 50%, similar to a class D hearing. The House Brackmann (HB) scale was used to rate facial nerve paresis [26]. Dizziness and/or unsteadiness complaints were collectively defined as vestibular symptoms. Hearing, presence of tinnitus, and facial and trigeminal nerve functions were evaluated ipsilateral to the side of the PRT-treated VS. Symptoms were evaluated at the most recent clinical contact.

Statistical analyses were performed in IBM SPSS Statistics for Windows (version 27.0. Armond, NY, USA: IBM Corp.). Descriptive statistics were used to evaluate patient characteristics and outcome variables.

## 3. Results

### 3.1. Patient Characteristics

Eight NF2 patients with a PRT-treated VS were identified and included in the analysis (Table 1). The median age was 36 years (IQR 20–66) and four patients (50%) were female. The NF2 phenotype was of the severe Wishart type in four patients, while the milder Feiling–Gardner type was detected in the remaining four patients. The indication for PRT was adjuvant treatment (*n* = 2, 25%), an increase in symptoms (*n* = 2), tumor progression (*n* = 1, 13%), brainstem compression (*n* = 1), and tumor recurrence (*n* = 1). The indication for PRT was not specified in one case. Fractionated PRT was administered to five patients (63%), while three (38%) received proton SRS. In each patient, only one VS was irradiated with proton therapy.

Prior to PRT, six patients (75%) received VS surgery for the target tumor, while one patient also underwent VS surgery twice for the contralateral VS. Three patients received bevacizumab targeted at the bilateral VS (Table 2). After PRT, three of six patients (50%) that were clinically followed up received additional treatments. Patient #1 and #5 received several courses of bevacizumab. In addition, patient #1 received a course of dasatinib and several courses of brigatinib for other CNS lesions. Patient #6 underwent placement of a ventriculoperitoneal shunt, and shortly thereafter a re-resection of the target tumor because of tumor progression.

Four cases were excluded from the present analysis as they did not meet the NF2 criteria. No patients were excluded due to the absence of follow-up data.

### 3.2. The Need for Salvage Therapy after PRT

**Figure 1:**
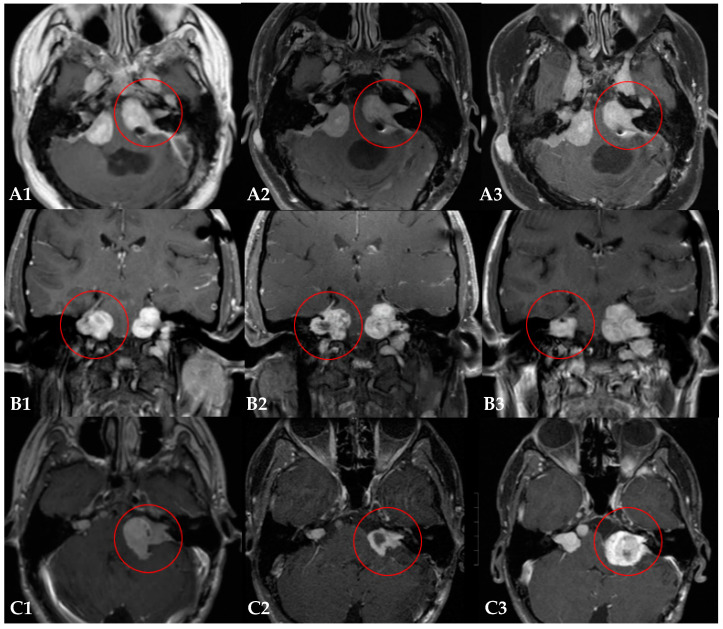
Three examples of the radiological follow-up of Neurofibromatosis Type 2-related vestibular schwannomas treated with proton radiotherapy (PRT). Each row (**A**–**C**) represents MR imaging of one patient over time: the baseline MR image (**1**), the MR image one year after PRT (**2**), and the most recent MR image (**3**). Patient (**A**): the tumor of interest remained stable during the follow-up of 60 months (**A1**–**A3**). Patient (**B**): the initial tumor shrinkage achieved with PRT and adjuvant bevacizumab therapy (**B1**), was followed by tumor progression (**B2**) that required additional treatments with bevacizumab (**B3**). Patient (**C**): the initial tumor shrinkage (**C1**,**C2**) was followed by significant tumor progression (**C3**), requiring salvage surgery at 87 months after PRT.

The median tumor volume was 4.2 cm^3^ (IQR 0.9–9.3) prior to PRT. The median radiologic follow-up after PRT was 71 months (IQR 62–85). Six out eight patients (75%) did not require salvage therapy after PRT for the target VS during the follow-up period (Table 3, Figure 1).

Two patients (25%) required additional treatment for tumor progression of the irradiated lesion. In patient #5, adjuvant bevacizumab therapy was required after the PRT, resulting in the initial tumor shrinkage of the irradiated tumor. Due to hearing loss and rapid tumor progression after stopping the adjuvant bevacizumab, bevacizumab was restarted 12 months after the PRT. Substantial tumor shrinkage (−59%) was observed under this course of bevacizumab three months later. During follow-up, patient #5 received several other courses of bevacizumab to maintain tumor control of the target VS (Figure 1, row B). In patient #6, surgical resection was required at 85 months (7 years) after the PRT, due to tumor progression (Figure 1, row C). Another patient (patient #1) received several courses of systemic therapies (bevacizumab, dasatinib, and brigatinib) after the PRT. However, in this case the irradiated tumor remained stable during follow-up and the systemic therapies were indicated for other lesions. The tumor of interest was therefore deemed free of progression after PRT.

Tumor pseudoprogression, defined as a short and self-limiting increase in tumor volume after radiation therapy, was evaluated in seven patients. Two of the seven patients (29%) experienced pseudoprogression at 2 and 7 months after the PRT, respectively.

### 3.3. Hearing

Six of the eight patients (75%) had no residual ipsilateral hearing prior to the PRT (Table 4).Three of these had lost hearing due the natural progression of the disease and three had suffered hearing loss after VS surgery (suboccipital (*n* = 2) and transpetrosal (*n* = 1) approaches).

Two patients (25%) with residual hearing prior to the PRT lost their hearing after radiation treatment. One patient (patient #3) had substantial hearing loss in the affected ear prior to the PRT (PTA: 92 dB, WRS: 0%; class D); ten months after the PRT, hearing had slightly decreased (PTA: 95 dB, WRS: 0%) until it was fully lost over time. Only one patient (patient #5) had near serviceable hearing before the PRT (PTA: 61 dB, WRS: 94% at 95 dB; class C); two months after the PRT, the PTA had dropped to 84 dB and the WRS to 28% at 107 dB (class D). Over the following two years, this patient’s hearing further decreased (PTA: 103 dB, WRS: 6% at 110 dB).

### 3.4. Treatment-Related Toxicity

Tumor and/or treatment-related morbidity was evaluated in six patients that were clinically followed after the PRT, as the other two patients’ charts only described the radiological follow-up (Table 4). The median clinical follow-up time was 71 months (IQR 66–83). At the baseline, five of the six patients (83%) had already reported symptoms: one (17%) patient had a mild facial paresis (HB 2), four reported the presence of tinnitus (67%), and four experienced vestibular symptoms. After the PRT, the facial nerve dysfunction worsened to a severe paresis (HB 5) in the patient with pre-existing paresis, while four patients (67%) developed new facial nerve dysfunctions: one patient (17%) experienced sporadic hemifacial spasms, another developed a moderately severe paresis (HB 4), two patients (33%) had severe paresis (HB 5), and one suffered from a total paralysis (HB 6). Trigeminal hypoesthesia developed in two patients after the PRT. Tinnitus remained stable in four patients, while two reported new onset tinnitus. Vestibular symptoms were unchanged in two patients, worsened in two patients, and occurred as a new onset in the remaining two patients.

## 4. Discussion

The majority of the NF2 patients who received PRT for VS (75%) in this cohort did not require local salvage therapy after treatment with PRT. However, tumor and/or treatment-related morbidity was substantial. The only two patients with residual hearing prior to the PRT lost their hearing during the follow-up period. As one of these patients did not receive additional treatment or experience tumor progression after the PRT, it appears that in this case the hearing loss was caused by the PRT. The other patient with residual hearing received several courses of bevacizumab after the PRT because the tumor had progressed. In this case, it thus remains unclear whether hearing loss was attributable to the toxic effects of the PRT or the result of tumor progression. The majority of patients also suffered from persistent, new onset, or increased pre-existing cranial nerve deficits. The pre-existing symptoms may be attributed to the natural progression of the disease or the earlier treatments received. After PRT, all patients that were clinically followed up experienced new onset or increased symptoms. While these may be the result of the PRT, it is worth noting that all patients received PRT as a salvage treatment. In addition, three patients (patient #1, #5, and #6) required additional interventions after the PRT. Interestingly, all three patients requiring additional therapy after the PRT suffered from the more aggressive ‘Wishart’ NF2 phenotype. Overall, one has to be cautious in linking the increased morbidity to PRT alone, rather it reflects the cumulative effect of PRT, the natural progression of the disease, and other prior or subsequent treatments..

To our knowledge, this is the first report on the clinical outcome and toxicity of PRT for VS in a cohort of NF2 patients to date; however, various studies have reported on PRT for sporadic VS [18,27]. In these studies, a combined total of 497 VS patients received passive-scatter PRT in a variety of treatment regimens between 1991 and 2018, with a prescribed dosage ranging from 12 to 60 Gy(RBE). Fifteen NF2 patients were identified within these study populations, but the clinical outcomes were only specified for one case. Compared to our cohort, the patients with unilateral, sporadic VS were older at time of treatment, underwent fewer VS-related therapies prior to PRT, had smaller tumor volumes at the start of treatment, and had better baseline hearing. The local tumor control after PRT in sporadic VS ranged from 85% to 100% (median follow-up 2.2–7.4 years), which is somewhat higher than in the present NF2 patient cohort, of whom 75% did not require salvage treatment. Additionally, we found more treatment-related toxicities in our NF2 cohort than were reported in sporadic VS: hearing loss occurred in 21% to 78% of the patients, facial nerve function loss in 0% to 10% of the patients, and trigeminal neuropathy was reported in 0% to 9% of the patients after PRT in sporadic VS. One study additionally reported on vestibular symptoms and tinnitus [18]. Severe dizziness was reported in 6%, while 29% experienced mild symptoms. Tinnitus was present in 83% of the patients. A recently published single-center study looked at both PhRT and PRT in VS in terms of tumor control, symptom evolution, and quality of life [28]. Eight NF2 patients (3%) were included in the analysis: one received photon SRS, four received fractioned PhRT, and three received fractioned PRT. The median follow-up was 38 months (3.2 years). Overall, tumor control and symptom evolution were found to be comparable across all three treatment regimes. At 12 months, a local tumor control rate of 99.5% was reported and cranial nerve deficits were limited. Nevertheless, it was found that patients with NF2-related VS were at a substantially greater risk of experiencing tumor progression, cranial nerve dysfunction, and vestibular symptoms compared to patients with a sporadic VS.

A possible explanation for the difference in local tumor control and treatment-related toxicity after PRT, between sporadic VS and the NF2-related VS, is that NF2-linked tumors may be less sensitive to radiation therapy, especially in the context of a more aggressive NF2 phenotype. This has indeed been demonstrated for PhRT [29,30]. In addition, the large majority of our study cohort had undergone surgery prior to PRT, which may cause pre-PRT function loss and/or increased vulnerability of structures surrounding the tumor.

Several studies have looked at the effect of PhRT in NF2 patients. A large systematic review including 485 NF2 patients with VS reported a sample-size weighted average of 75% tumor control after a mean follow-up duration of 5.2 years (SD 2.2; range 2.2–10.0 years) following photon SRS [31]. The reported average age and tumor volume were similar to the present cohort. While their reported tumor control was equal to the present cohort, preservation of hearing, and facial and trigeminal nerve function were markedly higher in the photon group. However, a significantly lower prior surgery rate of 23% was reported. A recently published systematic review and meta-analysis also covered the effect of photon SRS for VS in NF2 [13]. A total of 750 tumors were irradiated with a mean dose of 13.2 up to 25.0 Gy using a variety of radiation techniques. The reported patient characteristics were comparable to ours, except the rate of previous treatments—only a third of patients had received prior surgical resection. A radiological tumor control of 89% (mean follow-up 0.3–20.9 years) was achieved. After treatment, a clear decrease was reported for serviceable hearing (odds ratio [OR] 0.26), and increased risks of facial (OR 1.62) and trigeminal (OR 1.42) nerve dysfunction were found. These results agree with our findings after PRT.

The present analysis has several strengths. First, the patient sample is reflective of the diverse and complex patient population seen in NF2. Second, a wide range of NF2- and treatment-related symptoms were included in the chart review, allowing for a comprehensive evaluation. The study limitations include the small number of included patients (inherent to the rarity of the disease and the limited availability of PRT) and the retrospective study design. Furthermore, although the median duration of follow-up was nearly 6 years (71 months), which is adequate for reporting on short term tumor progression and toxicity, it may be insufficient to detect long-term sequelae, such as secondary tumors [20]. In addition, while radiologic follow-up was available in all cases, clinical follow-up was not available in two out of eight patients. Last, the reported toxicities after PRT should be interpreted with care as they are probably not solely attributable to PRT, but are the composite effect of the disease itself and the prior or adjuvant therapies.

Physicians are faced with difficult dilemmas when managing VS in NF2 patients [9,10,11]. The available active interventions—in addition to the sequelae of the disease itself—may have significant side effects and could result in the (further) losses of hearing, balance, and facial nerve and trigeminal nerve function. Active surveillance maybe the least harmful management option, at least initially, although the lack of intervention could result in the development of larger tumors and more severe disease-related symptoms, which may also require more extended and debilitating interventions. Especially in NF2, this dilemma is further complicated by the typical bilateral manifestation of VS, and the occurrence of concomitant intracranial tumors. As none of the current NF2 treatment modalities are without serious risks and/or sequelae, selecting the optimal treatment strategy, or the need for salvage therapy, is a challenge and is highly dependent on careful multidisciplinary evaluation [32]. In recent years, the use of radiotherapy to treat benign central nervous system tumors including VS has increased. However, especially in tumor susceptibility syndromes, such as NF2, there is a risk of malignant transformation of irradiated benign tumors and the development of new neoplasms in tissues surrounding the irradiated tumor [20,33]. Proton radiotherapy may be a valuable addition to the available treatment options in NF2 as it may reduce radiation in structures adjacent to the VS, and thus reduce toxicity and induction in secondary tumors and malignancy. The limited irradiation of surrounding normal tissue with PRT could also enable a safer future opportunity for additional radiation therapy of neighboring tumors.

## 5. Conclusions

The present study indicates that the majority of NF2 patients treated with PRT for VS did not require additional therapy. Similar to PhRT, the treatment-related toxicity was significant. A comparison of the current PRT data with the PhRT literature is flawed, however, because in the current cohort the PRT was deployed as a salvage treatment after surgery and/or after bevacizumab therapy, in the majority of patients. A longer follow-up of prospective NF2 cohorts with PRT as the primary treatment for VS is needed to critically evaluate its potential benefit. In addition, the potential of recent, more precise proton radiation delivery techniques, such as pencil beam scanning, require further evaluation.

## Figures and Tables

**Table 1:** Patient demographics and proton radiotherapy treatment plan.

No.	Age at Start of PRT (Years)	Sex	NF2 Phenotype	Treatment Side	PRT Dosage (Gy(RBE))	Fractions (*n*)	Indication for PRT
1	21	M	Wishart	L	52.7	31	adjuvant
2	44	M	Feiling–Gardner	R	50.4	28	recurrence
3	68	F	Feiling–Gardner	R	12.0	1	increasing symptoms
4	27	F	Wishart	L	54.0	30	tumor progression
5	19	M	Wishart	R	54.0	30	adjuvant
6	17	M	Wishart	L	54.0	30	brainstem compression
7	59	F	Feiling–Gardner	R	12.0	1	increasing symptoms
8	68	F	Feiling–Gardner	L	12.0	1	not indicated

Legend: no. = case number; PRT = proton radiotherapy; NF2 = neurofibromatosis type 2; Gy(RBE) = Gray—relative biological effectiveness; *n* = number; M = male; F = female; L = left; R = right.

**Table 2:** An overview of the treatments received in addition to proton radiotherapy.

No.	Surgery (Time to PRT)		Bevacizumab		Other	
	Before PRT	After PRT	Before PRT	After PRT	Before PRT	After PRT
1	yes (14 m)	no	yes	yes	no	dasatinib, brigatinib
2	yes (19 y)	no	no	no	no	no
3	no	no	no	no	no	no
4	yes (15 y)	no	yes	no	VPS, ABI	no
5	yes (5 m)	no	yes	yes	bortezomib	no
6 ^1^	yes (12 m)	yes (7 y)	no	no	no	VPS
7	yes (30 y)	n.i.	n.i.	n.i.	n.i.	n.i.
8	no	n.i.	n.i.	n.i.	n.i.	n.i.

^1^ surgical intervention defined as endpoint. Legend: no. = case number; PRT = proton radiotherapy; m = months; y = years; VPS = ventriculoperitoneal shunt; ABI = auditory brainstem implant; n.i. = not indicated.

**Table 3:** Tumor volumetry after proton radiotherapy.

No.	Volume at Baseline (cm^3^)	Volume at First FU after 1 Year (cm^3^)	Time to First FU (Months)	Volume at Most Recent FU (cm^3^)	Time to Most Recent FU (Months)	Need for Salvage Therapy (Yes/No)
1	17.71	13.8	15	11.44	60	no
2	5.36	5.31	16	3.19	59	no
3	0.57	0.55	21	0.44	69	no
4	2.96	2.44	13	2.75	80	no
5	8.7	7.05	12	3.52	68	yes
6 ^1^	9.46	4.0	17	13.79	87	yes
7	0.8	n.i.	n.i.	0.47	72	no
8	1.1	n.i.	n.i.	0.2	108	no

^1^ surgical intervention defined as endpoint. Legend: no. = case number; cm^3^ = cubic centimeters; FU = follow-up; n.i. = not indicated.

**Table 4:** The presence of symptoms and/or treatment-related toxicity during the clinical follow-up.

No.	1	2	3	4	5	6	7 ^1^	8 ^1^
Hearing								
Before PRT	deaf	deaf ^2^	loss	deaf ^2^	loss	deaf ^2^	deaf	deaf
After PRT	-	-	deaf	-	deaf	-	-	-
Facial nerve function (HB grade)								
Before PRT	1	1	1	1	2	1	n.i.	n.i.
After PRT	4	spasms ^3^	1	5	5	6	n.i.	n.i.
Trigeminal nerve function								
Before PRT	no	no	no	no	no	no	n.i.	n.i.
After PRT	hypo- esthesia	no	no	hypo- esthesia	no	no	n.i.	n.i.
Tinnitus								
Before PRT	yes	yes	yes	yes	no	no	n.i.	n.i.
After PRT	stable	stable	stable	stable	yes	yes	n.i.	n.i.
Vestibular symptoms								
Before PRT	yes	no	yes	yes	yes	no	n.i.	yes
After PRT	stable	yes	worse	worse	stable	yes	worse	n.i.
Other								
Before PRT	-	-	-	HCP	-	HA	n.i.	n.i.
After PRT	F	F	F, HD	-	-	HCP, HA	n.i.	n.i.
Time to most recent FU (months)	64	71	70	81	66	87 ^4^	72 ^1^	108 ^1^

^1^ only radiological follow-up available; ^2^ following surgical resection; ^3^ HB grade not indicated; ^4^ surgical intervention defined as the endpoint. Legend: no. = case number; PRT = proton radiotherapy; HB = House Brackmann; n.i. = not indicated; HCP = hydrocephalus; HA = headache; F = fatigue; FU = follow-up.

## Data Availability

The data presented in this study are available on request from the corresponding author. The data are not publicly available due to privacy restrictions.
